# Additive effect of knockdown resistance mutations, S989P, V1016G and F1534C, in a heterozygous genotype conferring pyrethroid resistance in *Aedes aegypti* in Thailand

**DOI:** 10.1186/s13071-016-1713-0

**Published:** 2016-07-26

**Authors:** Suriya Plernsub, Jassada Saingamsook, Jintana Yanola, Nongkran Lumjuan, Pongsri Tippawangkosol, Kom Sukontason, Catherine Walton, Pradya Somboon

**Affiliations:** 1 Department of Parasitology, Faculty of Medicine, Chiang Mai University, Chiang Mai, Thailand; 2 Department of Medical Technology, Faculty of Associated Medical Sciences, Chiang Mai University, Chiang Mai, Thailand; 3 Research Institute for Health Sciences, Chiang Mai University, Chiang Mai, Thailand; 4 Faculty of Life Sciences, University of Manchester, Manchester, UK

**Keywords:** *Aedes aegypti*, *kdr*, Insecticide, Genetics

## Abstract

**Background:**

Mutation in the voltage-gated sodium channel gene that results in knockdown resistance (*kdr*), is a major mechanism of pyrethroid resistance in several mosquito species. In *Aedes aegypti*, V1016G (occurring with and without S989P) and F1534C mutations are common and widely distributed throughout Asia. The G1016 allele is known to be associated with resistance to type I and II pyrethroids. The C1534 allele is primarily associated with resistance to type I pyrethroids and is known to be a recessive allele in conferring *kdr*.

**Methods:**

We performed crossing experiments using a P989 + G1016 homozygous mutant strain (UPK-R), a C1534 homozygous mutant strain (PMD-R) and a pyrethroid susceptible strain (PMD) to determine the insecticide susceptibility of different genotypic hybrids. Allele-specific PCR methods were used to confirm the genotypes. Metabolic resistance caused by oxidative enzymes and esterase enzymes was ruled out by the addition of piperonyl butoxide (PBO) and bis(4-nitrophenyl)-phosphate, BNPP), respectively.

**Results:**

The median lethal concentration (LC50) of deltamethrin susceptibility of a S/P989 + V/G1016 + F/F1534 double heterozygous hybrid from the UPK-R × PMD cross was 0.57 (95 % CI: 0.51–0.63) μgl^-1^, which was about 12-fold lower than for UPK-R, 6.98 (6.10–8.04) μgl^-1^, and only about 4-fold greater than the susceptible PMD, 0.13 (0.12–0.15) μgl^-1^. This resistance returned to 0.08 (0.07–0.09) μgl^-1^ on the addition of PBO suggesting that the P989 + G1016 *kdr* alleles are recessive. The LC50 of the S/P989 + V/G1016 + F/C1534 triple heterozygous hybrid was 3.58 (3.21–3.95) μgl^-1^, which was intermediate between that of the homozygous mutant genotypes, being 2-fold higher than the C1534 homozygote and 2-fold lower than the P989 + G1016 homozygote. These minor differences and the high LC50 values of the triple mutated heterozygote indicate there is some degree of functional equivalence of the P989 + G1016 and C1534 alleles in the heterozygote. Addition of PBO decreased the LC50 values by 2-fold, from 3.58 (3.21–3.95) to 1.52 (1.35–1.73) μgl^-1^, suggesting that oxidase enzymes play a partial role in resistance. The results are consistent with the median lethal time (LT50) of the triple mutated heterozygote against 0.05 % deltamethrin paper. An adult susceptibility test also revealed that the triple mutated heterozygote was resistant to deltamethrin and permethrin.

**Conclusions:**

The combination of the three *kdr* alleles in the triple mutated heterozygote, S/P989 + V/G1016 + F/C1534, confers high resistance to pyrethroids. This heterozygous form is common in *Ae. aegypti* populations throughout Thailand and may have an adverse effect on the efficacy of a mosquito control program using insecticide-based approaches.

## Background

*Aedes aegypti* is the primary vector of dengue virus and other viral diseases such as yellow fever, chikungunya and Zika, which are all public health problems in many countries throughout the world [1, 2]. Since human vaccines are not available, except for yellow fever, control of transmission is necessarily based on the management of breeding places, or the application of larvicidal (e.g. temephos sand granules) and adulticidal chemicals (e.g. fogging and ultra-low-volume sprays). The adverse effect of the heavy and long-term use of insecticides is resistance of *Ae. aegypti* worldwide. Insecticide resistance in *Ae. aegypti*, as well as in other vectors and pest species, is generally conferred by two major mechanisms: metabolic enzyme-based resistance and target site insensitivity [3]. Metabolic enzyme-based resistance is principally associated with three enzyme groups: cytochrome P450 monooxygenases (P450s), esterases and glutathione-S-transferases, depending on the insect species/strain and the insecticide. This mechanism can be reduced or inhibited by metabolic inhibitors, such as piperonyl butoxide (PBO) and bis(4-nitrophenyl)-phosphate (BNPP), a monooxygenase inhibitor and an esterase inhibitor, respectively. Target site insensitivity in mosquitoes and other insects is associated with single or multiple mutations of the voltage-gated sodium channel (VGSC) protein, commonly referred to as knockdown resistance (*kdr*). These mutations modify the VGSC protein, making it less susceptible to the binding of pyrethroids and DDT [4]. This mechanism is not overcome by metabolic inhibitors.

Several mutations in VGSC of *Ae. aegypti* have been reported, but only a few of them have been confirmed to be associated with pyrethroid resistance. A valine to glycine transversion in domain II of the VGSC (V1016G) is associated with resistance to type I and II pyrethroids, such as permethrin and deltamethrin, respectively [5]. The V1016G mutation appears to be restricted to Asia [6–15]. A second mutation, involving a phenylalanine to cysteine substitution at position 1534 within domain III (F1534C), is associated with resistance to type I pyrethroids [16]. The F1534C mutation is widely distributed in Asia [8, 9, 11–15, 17–20] and Latin America [21–24]. Moreover, a valine to isoleucine transversion in domain II (V1016I), conferring pyrethroid resistance, occurs among *Ae. aegypti* populations in Latin America [22–25].

The V1016G mutation is often found with a serine to proline mutation (S989P) in domain II, but S989P has not been found alone [9–12]. The S989P mutation was not detected in *Ae. aegypti* from Taiwan [7] or Malaysia [13], but in Thailand the V1016G mutation appears to always co-occur with S989P [10, 11]. Neurophysiological studies [26, 27] using the *Xenopus* oocyte expression systems revealed that the V1016G mutation reduces both permethrin and deltamethrin sensitivity, whereas the F1534C mutation reduces only permethrin sensitivity. The S989P mutation has no effect on permethrin sensitivity on its own or in combination with the V1016G mutation. Du et al. [26] did not find any additive effect of V1016G + S989P mutations on deltamethrin sensitivity. However, Hirata et al. [27] revealed that the V1016G + S989P mutation combination greatly reduced the channel sensitivity to deltamethrin. In addition, they found a far greater reduction of permethrin and deltamethrin sensitivity in S989P + V1016G + F1534C triple mutants expressed in oocytes (which are effectively homozygous) indicating a synergistic effect of the combination of mutant alleles. In wild populations of *Ae. aegypti*, the double homozygote, V1016G (with or without S989P) + F1534C mutations, were rare or absent [9, 11, 13, 15], which is probably due to a fitness cost or lack of recombination to date to bring the V1016G + S989P and F1534C mutations onto the same haplotype. In Malaysia, Ishak et al. [13] reported an additive effect to deltamethrin resistance in the double heterozygote mutant (V/G1016 + F/C1534) *Ae. aegypti* mosquitoes. However, the role of mixed function oxidases which appeared to be the major resistance mechanism was not ruled out. In *Ae. aegypti* populations in Thailand, S/P989 + V/G1016 + F/C1534 triple heterozygous mosquitoes were common and were more tolerant to deltamethrin than C1534 homozygous mosquitoes [11, 28]. Moreover, since *kdr* genes in *Ae. aegypti* are autosomal recessive [16, 25]; it is not clear whether this observed resistance is due only to *kdr* alleles or results in part from metabolic resistance, which also plays a role in pyrethroid resistance [29–31]. The present study employed crossing experiments to determine the resistance profile of heterozygous *kdr* allelic combinations, conferring deltamethrin resistance; while metabolic enzymes were ruled out by adding enzyme inhibitors.

## Methods

### Mosquito strains

Three strains of *Ae. aegypti*, namely PMD, PMD-R and UPK-R, which were established in our laboratory were used in this current study. PMD and PMD-R were established from field caught mosquitoes from Ban Pang Mai Daeng, Mae Taeng District, Chiang Mai Province since 1997. The PMD is susceptible to pyrethroids, but resistant to DDT conferred mainly due to increased DDTase activity [29]. No *kdr* mutations (S989P, V1016G and F1534C) are found in this strain (S/S989 + V/V1016 + F/F1534, or hereafter SS + VV + FF) [16]. The PMD-R, (S/S989 + V/V1016 + C/C1534, or SS + VV + CC), is homozygous for C1534, lacks S989P and V1016G mutations, and is resistant to both DDT and permethrin but susceptible to deltamethrin [16, 30, 31]. UPK-R was established from Chiang Mai city in 2006. It is homozygous for the G1016 allele and resistant to DDT, permethrin and deltamethrin. Our investigations revealed that the S989P homozygous mutation is also found in all individuals of this strain. Therefore, UPK-R harbors P/P989 + G/G1016 + F/F1534, or PP + GG + FF. The permethrin resistance level of UPK-R and PMD-R was higher than the susceptible PMD by 325-fold and 25-fold, respectively, as determined by larval bioassays. Mixed function oxidases play a partial role in pyrethroid resistance in PMD-R and UPK-R ([16, 29–31] and P. Somboon, unpublished data). PMD-R and UPK-R adult mosquitoes were maintained under regular insecticide pressure (0.75 % permethrin and 0.05 % deltamethrin, respectively). The occurrence of the F1534C and V1016G mutations in the parental colonies was regularly checked by DNA sequencing and allele-specific PCR (AS-PCR) methods [11, 17]. The rearing of mosquitoes followed our routine procedures as previously described [11]. Blood meals were provided with an artificial membrane feeding method with cow blood [32], with minor modifications.

### Crossing experiments

Batches of blood fed female mosquitoes were taken from the stock colonies of each strain. They were kept separately in a cup lined with filter paper. Five days post-feeding, distilled water was added into the cups for oviposition. The laid eggs were allowed to stand for 4 days and then air-dried until used. To obtain the larvae, the eggs were submerged in distilled water, by which the larvae were readily hatched in 24 h. They were reared as described above until pupation. The pupae were sexed by examination of the genital lobe under stereomicroscope and kept separately until emergence. Since there are no maternal effects or sex linkage regarding *kdr* [16], a one directional cross was performed. Five days after emergence, crosses between UPK-R and the susceptible PMD and of UPK-R and the permethrin resistant PMD-R were carried out by allowing them (100 pairs of males and females for each cross) to mate freely in a 30 cm^3^ cage for 3 days. A blood meal was provided via membrane feeding as described above. After 5 days post-feeding an oviposition cup, lined with filter paper, containing distilled water was inserted into the cage for a few days. The laid hybrid eggs (F1) were removed and air dried until used. A number (at least 10) of fourth instar larvae reared from these eggs were selected at random to confirm their genotypes using DNA sequencing and AS-PCR methods as above.

### Insecticide susceptibility tests

Larval and adult susceptibility tests were conducted according to WHO standard methods [33, 34]. For larval bioassays, stock and serial dilutions of deltamethrin (99.5 % technical grade, Supelco, Bellefonte, PA, USA) were prepared in ethanol. The bioassays were conducted in 400 ml beakers containing 250 ml of distilled water and one of 5–7 different insecticide concentrations (0.03–500 μg/l) giving 0–100 % mortality. There were 4 replicates per concentration. The ethanol content in each assay solution was limited to 0.4 %. Batches of 25 early fourth-instar larvae were tested per beaker. In the control experiments, ethanol was included to a concentration of 0.4 % in 250 ml of water. In parallel with this, an extra set of bioassays with the addition of enzyme inhibitors, either piperonyl butoxide (PBO) (90 % technical grade, Aldrich, St. Louis, MO, USA) or bis (4-nitrophenyl)-phosphate (BNPP) (99 % technical grade, Aldrich, St. Louis, MO, USA), 0.3 mg/ml each, were performed to determine if the resistance was conferred by oxidase or esterase enzymes, respectively [35]. Larval mortality was recorded 24 h after exposure. Mortality data were corrected by natural control mortality using Abbott’s formula [36]. The concentration-mortality responses and the median lethal concentration (LC50) were determined by probit analysis [37] using the software LdP Line (LdP Line, copyright 2000 by Ehab MostofaBakr, Cairo, Egypt).

In the adult bioassay test, batches of about 25 non-blood fed females, 1–2 day-old, were exposed to 0.05 % deltamethrin and 0.75 % permethrin impregnated papers (WHO, Malaysia) for 60 min in standard WHO test tubes. Control mosquitoes were exposed to papers without insecticide. The test mosquitoes and the controls were held for a 24-h recovery period and the mortality recorded.

### Determination of median lethal time (LT50)

Batches of 25 1–5 day-old females were exposed to the 0.05 % deltamethrin paper at five different exposure time periods. The test mosquitoes and the controls were held for a 24-h recovery period and the mortality recorded. Four replicates were undertaken. Percentage mortalities were calculated for each exposure time and the mortality data was analyzed on a log-time probit mortality regression, as above.

## Results

Data from larval deltamethrin susceptibility tests of the parental strains and the F1 hybrids are presented in Table 1. The resistance ratio (RR) was calculated by comparison to the pyrethroid susceptible PMD strain. The LC50 values of deltamethrin susceptibility of the parental strains, UPK-R (PP + GG + FF), PMD-R (SS + VV + CC) and PMD (SS + VV + FF) were 6.98 (95 % confidence intervals, CI: 6.10–8.04), 1.72 (1.55–1.90) and 0.13 (0.12–0.15) μgl^-1^, respectively. Adding the oxidase inhibitor (PBO) decreased the LC50 values of UPK-R and PMD-R by 2-fold (based on non-overlap of their 95 % CI), suggesting that oxidative enzymes play a partial role in resistance in both strains. By contrast, adding the esterase inhibitor (BNPP) to the UPK-R and PMD-R bioassays had little or no effect on the LC50 values (reduction by 1.01- and 1.19-fold, respectively), suggesting that esterase enzymes play effectively no role in resistance. The UPK-R × PMD cross produced F1 hybrids that are heterozygous for mutant alleles at positions 989 and 1016, i.e. S/P989 + V/G1016 + F/F1534, or SP + VG + FF. The LC50 of this form was about 12-fold lower than the resistant UPK-R and only about 4-fold greater than the susceptible PMD. When PBO was added to eliminate the oxidase enzyme activity, the LC50 value was substantially reduced, being even lower than that of the susceptible PMD strain. The UPK-R × PMD-R cross produced F1 hybrids heterozygous for mutant alleles at positions 989, 1016 and 1534, i.e. S/P989 + V/G1016 + F/C1534, or SP + VG + FC. The LC50 of this form was about 28-fold higher than the susceptible PMD; interestingly, this was intermediate between that of the homozygous mutant genotypes, being 2-fold higher than PMD-R and 2-fold lower than UPK-R. Adding PBO resulted in a decrease of LC50 by 2-fold, which is still higher than the susceptible PMD by 12-fold. Again, in the presence of BNPP, there was little effect on the LC50 value, indicating that esterases are not important in resistance in any of the resistant strains and their hybrids.

**Table 1:** Larval bioassay with deltamethrin in *Aedes aegypti* strains and hybrids

Strain^a^	*kdr* genotype	Synergist^b^	LC50 (95 % CI) μgl^-1^	RR^c^	Slope ± SD	*χ* ^2^	*P* ^d^
UPK-R	PP + GG + FF	–	6.98 (6.10–8.04)	53.69	2.05 ± 0.14	5.93 (*n* = 6, *df* = 5)	0.204
UPK-R	PP + GG + FF	BNPP	6.85 (6.26–7.44)	52.69	3.34 ± 0.29	6.42 (*n* = 5 *df* = 4)	0.092
UPK-R	PP + GG + FF	PBO	4.93 (4.24–5.70)	37.92	1.88 ± 0.14	7.82 (*n* = 6, *df* = 5)	0.098
F1 (PMD-R*f* × UPK-R*m*)	SP + VG + FC	–	3.58 (3.21–3.95)	27.54	3.12 ± 0.23	9.39 (*n* = 6, *df* = 5)	0.051
F1 (PMD-R*f* × UPK-R*m*)	SP + VG + FC	BNPP	3.44 (3.10–3.78)	26.46	3.15 ± 0.27	5.21 (*n* = 5, *df* = 4)	0.156
PMD-R	SS + VV + CC	–	1.72 (1.55–1.90)	13.23	3.02 ± 0.19	8.13 (*n* = 7, *df* = 6)	0.149
F1 (PMD-R*f* × UPK-R*m*)	SP + VG + FC	PBO	1.52 (1.35–1.73)	11.69	2.57 ± 0.17	9.22 (*n* = 6, *df* = 5)	0.055
PMD-R	SS + VV + CC	BNPP	1.44 (1.31–1.59)	11.07	2.79 ± 0.19	3.85 (*n* = 6, *df* = 5)	0.426
PMD-R	SS + VV + CC	PBO	0.72 (0.63–0.81)	5.53	2.49 ± 0.23	3.86 (*n* = 6, *df* = 5)	0.275
F1 (UPK-R*f* × PMD*m*)	SP + VG + FF	–	0.57 (0.51–0.63)	4.38	2.47 ± 0.16	8.47 (*n* = 7, *df* = 6)	0.131
F1 (UPK-R*f* × PMD*m*)	SP + VG + FF	BNPP	0.48 (0.44–0.54)	3.69	2.67 ± 0.18	3.29 (*n* = 7, *df* = 6)	0.510
PMD	SS + VV + FF	–	0.13 (0.12–0.15)	1	3.09 ± 0.25	1.45 (*n* = 5, *df* = 4)	0.690
F1 (UPK-R*f* × PMD*m*)	SP + VG + FF	PBO	0.08 (0.07–0.09)	0.62	3.28 ± 0.24	8.57 (*n* = 6, *df* = 5)	0.727

^a^ *f* female, *m* male

^b^ *PBO* piperonyl butoxide, *BNPP* bis(4-nitrophenyl)-phosphate

^c^Resistance ratio is based on comparison with the PMD strain

^d^All *P*-values > 0.05 indicating that the plots of concentration-mortality response are normally distributed, and hence the LC50 values are valid

Table 2 shows the median lethal time (LT50) and susceptibility of the parental strains and F1 hybrids to deltamethrin and permethrin. The LT50 of the susceptible PMD was shortest whereas that of the resistant UPK-R was the longest but the exact value could not be obtained, since exposure ended at 3 h without knockdown and mortality being observed. The LT50 of PMD-R was about 10-fold longer than the susceptible PMD. The LT50 of the UPK-R × PMD-R hybrid (SP + VG + FC) was longer than the parental PMD and PMD-R, 55-fold and 5-fold, respectively, and was longer than the UPK-R × PMD hybrid (SP + VG + FF) by 9-fold. The data is comparable to the LC50 values in the larval bioassay in Table 1. Adult susceptibility tests revealed that PMD and the UPK-R × PMD hybrid (SP + VG + FF) were susceptible to deltamethrin and permethrin. PMD-R was resistant to only permethrin. Like UPK-R, the UPK-R × PMD-R hybrid (SP + VG + FC) was resistant to deltamethrin and permethrin.

**Table 2:** Median lethal time (LT50) and adult susceptibility test of *Ae. aegypti* strains and hybrids

Strain^a^	*kdr* genotype	LT50^b^ (min)	0.05 % Deltamethrin		0.75 % Permethrin	
		(95 % CI)	*n*	% mortality	*n*	% mortality
PMD	SS + VV + FF	2.31 (1.49–3.72)	105	100	105	100
F1 (PMD*f* × UPK-R*m*)	SP + VG + FF	13.54 (12.05–15.59)	105	100	105	98.0
PMD-R	SS + VV + CC	24.67 (22.69–26.69)	100	100	100	0
F1 (PMD-R*f* × UPK-R*m*)	SP + VG + FC	126.04 (117.23–134.10)	100	14.0	100	1.0
UPK-R	PP + GG + FF	> 180	109	0	105	0

^a^ *f* female, *m* male

^b^after exposure to 0.05 % deltamethrin

## Discussion

The current study clearly demonstrates that deltamethrin resistance in the UPK-R strain is conferred mainly by *kdr* mutations; the oxidative enzyme system plays a partial role in deltamethrin resistance and there is a negligible contribution from esterases. This is similar to the situation in the permethrin resistant PMD-R strain [16, 29–31]. Whereas these previous studies revealed that the C1534 mutation in the homozygous form is the major mechanism for permethrin resistance in the PMD-R strain. This study shows that deltamethrin resistance is conferred mainly by the P989 + G1016 mutation combination in the homozygous form. Our bioassay results confirm the neurophysiological studies [26, 27] that the V1016G mutation confers resistance to deltamethrin and permethrin; whereas, the F1534C mutation confers resistance to permethrin. In addition, deltamethrin sensitivity in the homozygous form of the P989 + G1016 mutation combination was 5-fold lower than that induced by the G1016 homozygote [27].

Mosquitoes that were heterozygous for only the 989 + 1016 *kdr* mutations (SP + VG + FF), derived from the cross between the resistant UPK-R and the susceptible PMD, had an LC50 only 4-fold greater than the susceptible PMD (SS + VV + FF). We attribute this low level of resistance primarily to the oxidative enzyme system since resistance is removed by the addition of the oxidase inhibitor, PBO. This therefore indicates that G1016 in the presence of P989 is a recessive allele in conferring *kdr*. Yanola et al. [16] similarly demonstrated that C1534 is a recessive allele for *kdr* as the small level of resistance in the F/C1534 heterozygote (derived from the PMD × PMD-R cross) was removed on addition of PBO. Saavedra-Rodriguez et al. [25] also demonstrated that the I1016 allele is recessive. Consequently, if any of these mutations is present as a single copy in mosquitoes it provides little or no resistance to pyrethroids.

Given the above, it is interesting that the combination of single *kdr* alleles at sites 989, 1016 and 1534, i.e. the triple heterozygous state S/P989 + V/G1016 + F/C1534, gives a relatively high level of resistance (Tables 1 and 2). This level of resistance, as indicated by the LC50 values, was intermediate between that of the homozygous mutant genotypes, being 2-fold higher than the C1534 homozygote (PMD-R) and 2-fold lower than the P989 + G1016 homozygote (UPK-R). This supports the findings of Ishak et al. [13] of an additive effect between the G1016 and C1534 *kdr* alleles in the heterozygote V/G1016 + F/C1534 (in the absence of S989P mutations) for deltamethrin resistance compared with the C1534 homozygote.

This additive effect contrasts with the finding of synergy between the P989 + G1016 + C1534 alleles for deltamethrin and permethrin resistance when they are effectively homozygote, i.e. when they are expressed from a triple mutated gene cloned into *Xenopus* oocytes [27]. In this situation, as a monomeric protein, all VGSCs in the cell will contain all three mutations. By contrast, because the P989 + G1016 and C1534 *kdr* alleles are on different haplotypes, in the triple mutated heterozygote each VGSC will contain either the P989 + G1016 or the C1534 *kdr* allele. This likely underlies the synergistic effect of the three mutations in the homozygote as computer models have indicated that pyrethroids preferentially bind to the open state of VGSC by interacting with two receptor sites formed by the interfaces of the transmembrane helix S6 of domains II and III. Simultaneous binding of pyrethroids to S6 in both domains II (e.g. position 1016) and III (e.g. position 1534) is necessary to efficiently lock sodium channels in the open state [26, 38]. However, further study is required to understand the synergistic mechanism.

Both the V1016G and F1534C *kdr* mutations have been found in *Ae. aegypti* populations throughout southeast Asia. In Thailand, the S/P989 + V/G1016 + F/C1534 triple mutated heterozygote was as common as the C1534 homozygote (about 45 % each), whereas the P989 + G1016 homozygote was found at about 10 % in *Ae. aegypti* populations [11, 28]. Our previous experiment with thermal fogging spray outdoors with deltamethrin + S-bioallethrin + PBO revealed that P989 + G1016 homozygous mosquitoes suffered no mortality and about half of the triple mutated heterozygous mosquitoes survived the spray. By contrast, most of C1534 homozygous mosquitoes were killed [28]. In natural conditions, the efficacy of thermal fogging spray is likely to be even less effective. Therefore, we emphasize the significant impact of P989 + G1016 homozygous and triple heterozygous S/P989 + V/G1016 + F/C1534 mutants on *Ae. aegypti* control programs using pyrethroid-based approaches. Of particular concern is the possibility that a highly resistant homozygous triple mutant haplotype, P989 + G1016 + C1534, may arise in natural populations by recombination. The lack of this genotype at an appreciable frequency to date is likely due to its low fitness as suggested in Hirata et al. [27]. However, it is possible that compensatory mutations that restore fitness could allow this genotype to proliferate which would make pyrethroids completely ineffective. This emphasizes the need for alternative methods or chemicals that can overcome *kdr* in the long term and the necessity for continued monitoring of resistance genotypes in *Ae. aegypti* populations.

## Conclusions

Both S989P + V1016G and F1534C mutations exist in *Ae. aegypti* populations in Thailand and contribute to the major mechanism of pyrethroid resistance, whereas oxidase enzymes have a partial role in resistance. The homozygous mutants actually exhibit high resistance to deltamethrin and/or permethrin. However, the combination of the three *kdr* alleles in the triple mutated heterozygote, S/P989 + V/G1016 + F/C1534, confers high resistance to pyrethroids. Both mutant homozygotes and the triple mutated heterozygotes are all present at high frequencies in Thailand. Continued monitoring of *kdr* mutations, fitness and pyrethroid resistance levels is essential.

## Abbreviations

AS-PCR, allele-specific PCR; BNPP, bis(4-nitrophenyl)-phosphate; C1534, cysteine allele at position 1534; CI, confidence intervals; F1, first generation of progeny; F1534, phenylalanine allele at position 1534; F1534C, phenylalanine to cysteine substitution at position 1534; G1016, glycine allele at position 1016; *kdr*, knockdown resistance; LC50, median lethal concentration; LT50, median lethal time; P450s, cytochrome P450 monooxygenases; PBO, piperonyl butoxide; PP + GG + FF, P/P989 + G/G1016 + F/F1534; RR, resistance ratio; S989P, serine to proline mutation at position 989; SP + VG + FC, S/P989 + V/G1016 + F/C1534; SP + VG + FF, S/P989 + V/G1016 + F/F1534; SS + VV + CC, S/S989 + V/V1016 + C/C1534; SS + VV + FF, S/S989 + V/V1016 + F/F1534; V1016, valine allele at position 1016; V1016G, valine to glycine transversion at position 1016; V1016I, valine to isoleucine transversion at position 1016; VGSC, voltage-gated sodium channel
